# Suggestion as a Factor in Causing Neuroses and Psychoses

**Published:** 1911-03

**Authors:** W. T. Marrs

**Affiliations:** Peoria Heights, Ill.


For Texas Medical Journal.

### Suggestion as a Factor in Causing Neuroses and Psychoses.

BY W. T. MARRS, M. D., PEORIA HEIGHTS, ILL.

This caption sounds a trifle ponderous, but I can assure you that my brief consideration of it will be in a very simple manner. We are all very conversant with the fact that many of the neurotic and functional ailments which we treat are amenable to suggestion in some form, and that without this psychic curative element being in some way aroused our medicines and remedial procedures would be almost nil. But we often overlook the fact that many of the ailments about which we are consulted are brought about through suggestive influences brought to bear on the sufferer's mind.

Women are peculiarly susceptible to suggestive influences, whether good or bad. Many of the ailments of womankind are of psychic origin, and not infrequently the doctor's line of talk, always intended to be helpful, only puts new disease ideas in the patient's head. This is not Christian Science, either. The woman who has a slight cervical laceration, leucorrhea, endometritis, anteversion or retroversion of the uterus, or other condition, mild or grave, and is strongly impressed by what her physician tells her of the particular infirmity, always intensifies the trouble by her introspective attitude toward it. In a grim sort of way a woman who has an inherent tendency toward neurasthenia or mental instability loves to dilate on her ailments, and in a little while she quite naturally finds herself growing worse. Hysteria is often manifested in no other way only by the attention a woman may desire addressed to her ailments. Several years ago I was called a long distance to see a woman of 50 who had long been a sufferer from a chain of symptoms, all neurotic in character, and this time her manifestations were decidedly on the cyclonic order. For several days she had, according to her own version of the case, been passing "great chunks of flesh from her bladder." The attendants and friends were greatly exercised about this. I could discover no evidence of bladder, urethral or kidney impairment, and the attending physician said that none such existed. Upon careful inquiry, we learned that a lady in the neighborhood had suffered from a cystitis, and the knowledge of the case had come to this woman, after which she worked up a beautiful case of "bladder trouble" of her own. Just at this time I am treating a neurotic woman of about 55 who tells me that she has abscesses on her liver and that she is passing with her urine great quantities of pus and "great chunks of green matter." Scrutinizing the case for a day or so to verify this report, I find that there is nothing to it and that the woman had heard recently of a case of abscess of the liver.

An extreme case of hysteria in a young girl came under my observation a few years ago with all the protean and far-reaching symptoms which that malady affords. One stunt which she pulled off for a few weeks was that of hydrophobia. She could bark like a dog most beautifully, snapped at her friends, frothed at the mouth, lapped her drink, and so on *ad nauseam*. Upon thorough inquiry and research, I found that this idea of hydrophobia had been put in her head by a physician casually remarking that a certain ocular symptom was "photophobia." She took her cue from this and misinterpreting the term worked up a fine case of the other.

When a nervous and impressionable woman gets the idea of an operation firmly entrenched in her mind, little short of a surgical intervention will relieve her symptoms, whether an operation be indicated or not. A great deal of the "painful ovaries" and "weak back from female weakness" are simply due to over-attention to these organs and their functions. The athletic girls who forget that they have "wombs" and other ill-behaved organs suffer less in this direction than those who so often feel constrained to carefully guard their health and indulge in continuous treatment. The nostrum venders who exploit female strengtheners and local treatments to the sex do much to perpetuate neuroses in women. As a matter of fact, the women with grave abnormalities, such as a badly prolapsed uterus and horribly split-up cervix, do not as a rule make so much fuss about these conditions as do the nervous females with slight lesions, all things, of course, being equal.

Doctors should, therefore, exercise the greatest discretion and care, lest they suggest conditions that the woman does not already suffer seriously from, or augment those already present. It is a hard matter for a woman of weak nerves and lowered inhibition to get the idea of disease out of her mind when it has reference to any of her important organs, sexual or otherwise. Some weeks ago I was consulted by a young lady, a school teacher, who suffered from "slight appendicitis and a diseased condition of a *portion* of one ovary." Pretty fine diagnosis that. But it was what a lady physician had told the patient, and an operation seemed the only alternative in the minds of patient and physician. The young lady was sick, moping and despondent beyond measure, all because of the operation which was in the end, of course, to woo her back to health and happiness. But she dreaded the ordeal of entering the operating arena. In a ruthless and almost iconoclastic spirit, I said: "There is no appendicitis, at least not in your case, and operable cases of ovarian disease in young women seldom exist, and, when they do, they are almost invariably of venereal origin." She was admonished regarding both her physical and mental health, and urged to forget that she possessed organs, healthy or otherwise. There was a rapid amelioration of her symptoms and in a surprisingly short time the lady had forgotten that she was an invalid.

Scores of cases might be cited to illustrate that much from which women suffer are simply neuroses, originating in their own morbidly introspective minds or suggested by friends, physicians or nostrum literature. Of course, in this connection, I have reference only to those conditions which are not supported by real pathological conditions. Of the latter, we know there is an abundance. I was also going to speak of the neurasthenic states in men, which are brought about by adverse suggestive influence, but this paper has already gained sufficient length.

